# Tumor necrosis factor-alpha induced expression of matrix metalloproteinase-9 through p21-activated Kinase-1

**DOI:** 10.1186/1471-2172-10-15

**Published:** 2009-03-19

**Authors:** Ling Zhou, Chunli Yan, Roben G Gieling, Yujiro Kida, Warren Garner, Wei Li, Yuan-Ping Han

**Affiliations:** 1 Keck School of Medicine, University of Southern California, Los Angeles, CA 90033, USA

## Abstract

**Background:**

Expressed in embryonic development, matrix metalloprotein-9 (MMP-9) is absent in most of developed adult tissues, but recurs in inflammation during tissue injury, wound healing, tumor formation and metastasis. Expression of MMP-9 is tightly controlled by extracellular cues including pro-inflammatory cytokines and extracellular matrix (ECM). While the pathologic functions of MMP-9 are evident, the intracellular signaling pathways to control its expression are not fully understood. In this study we investigated mechanism of cytokine induced MMP-9 with particular emphasis on the role of p21-activated-kinase-1 (PAK1) and the down stream signaling.

**Results:**

In response to TNF-alpha or IL-1alpha, PAK1 was promptly activated, as characterized by a sequential phosphorylation, initiated at threonine-212 followed by at threonine-423 in the activation loop of the kinase, in human skin keratinocytes, dermal fibroblasts, and rat hepatic stellate cells. Ectopic expression of PAK1 variants, but not p38 MAP kinase, impaired the TNF-alpha-induced MMP-9 expression, while other MMPs such as MMP-2, -3 and -14 were not affected. Activation of Jun N-terminal kinase (JNK) and NF-kappaB has been demonstrated to be essential for MMP-9 expression. Expression of inactive PAK1 variants impaired JNK but not NF-kappaB activation, which consequently suppressed the 5'-promoter activities of the MMP-9 gene. After the cytokine-induced phosphorylation, both ectopically expressed and endogenous PAK1 proteins were promptly accumulated even in the condition of suppressing protein synthesis, suggesting the PAK1 protein is stabilized upon TNF-alpha stimulation. Stabilization of PAK1 protein by TNF-alpha treatment is independent of the kinase catalytic activity and p21 GTPase binding capacities. In contrast to epithelial cells, mesenchymal cells require 3-dimensional type-I collagen in response to TNF-alpha to massively express MMP-9. The collagen effect is mediated, in part, by boost JNK activation in a way to cooperate the cytokine signaling.

**Conclusion:**

We identified a novel mechanism for MMP-9 expression in response to injury signals, which is mediated by PAK1 activation and stabilization leading JNK activation.

## Background

Degradation of extracellular matrix (ECM), as mediated by matrix metalloproteinases (MMPs) and antagonized by tissue inhibitors of matrix metalloproteinases (TIMPs), is critical for embryonic development and adult tissue homeostasis [1,2]. Conversely, uncontrolled ECM degradation occurs in many degenerative diseases [3,4]. For instance, excessive presence of MMPs has been well documented in cancer dissemination, arthritis development, and chronic wound progression [5]. Among the twenty-some members of the MMP family in either the human or murine genome the proteinases can be classified not only based on their structural conservation, but also by the means of their expression. Of our interest for many years is the regulation of MMP-9, a type-IV collagenase in tissue injury and repair. Not expressed in most of adult developed tissues, MMP-9 is promptly expressed in response to tissue damages under the control of pro-inflammatory cytokines [6,7]. Nascent proMMP-9 is maintained in latency through interaction between a conserved cysteine in the pro-peptide domain and a zinc atom in the catalytic domain. Furthermore, TNF-α-induced maturation of proMMP-9 by skin cells is mediated by down regulation of TIMP-1, while the converting enzymes are seemingly constitutively present [8]. Recently we uncovered alpha-1-antichymotrypsin (alpha-ACT) as a novel pathological inhibitor that directly antagonizes the proMMP-9 converting enzyme in skin tissues [9]. Thus, generation of active MMP-9 within tissues is tightly monitored at the levels of gene expression, protein processing, and antagonization by inhibitors targeting at either proMMP-9 (by TIMP-1) or converting enzyme (by alpha-ACT).

Innate immunity and its produced waves of inflammatory cytokines are the initial signals to trigger expression of MMPs in tissue injury phase. Compelling evidence of *in vitro *experiments also demonstrates the critical roles of inflammatory cytokines in control of MMP expression and activation. Intracellular signals governing the expression of MMPs have been extensively studied; among them, Jun N-terminal kinase (JNK) and nuclear factor kappa B (NF-kB) signaling pathways are essential to induce many MMPs [10,11]. Still outstanding is that how other extracellular cue, such as ECM, in cooperation with pro-inflammatory cytokines control MMP expression, of which is particularly critical for mesenchymal cells. For instance, in addition to TNF-α or IL-1, the 3-dimentional type-I collagen is also required to maximally induce MMP-9 by either human dermal fibrobaslts or rat hepatic stellate cells [6,7].

p21-activatd kinase (PAK), a family of serine/threonine kinases conserved from yeast to human, is important for regulation of cytoskeleton, cell migration, and cell cycle progression [12]. PAK1 was originally identified through its binding to p21 GTPase [13,14]. Like many other protein kinases, PAK1 retains inactive by its own pseudo-substrate like domain, and is activated by binding GTP-charged proteins, which consequently leads conformational changes and auto-phosphorylation. Non-canonically, PAK1 can be phosphorylated and activated by PDK1 which phosphorylates a conserved threonine-423 within the active loop [15]. In addition to regulating cytoskeletal proteins, PAK1 also controls MAP kinases, such as JNK and p38 MAP kinases [16,17]. Furthermore, PAK1 also modulate NF-κB activity [18,19]. Although the dynamics of cytoskeleton and the formation of stress filaments are critical for cell-matrix interactions in inflammation, very little is known if and how inflammatory cytokines regulate PAK1 in control of MMP expression.

In this study we demonstrated an unrecognized mechanism by which TNF-α activates PAK1 by a sequential phosphorylation from threonine-212 to threonine-423 in human epithelial cells and fibroblasts. Similarly, IL-1α also promotes PAK1 activation in rat hepatic stellate cells. Moreover, TNF-α treatment promptly results in accumulation of PAK1 protein, but not p38 MAP kinase, in part by stabilization of the former. Although the details are unknown to date, TNF-α-mediated stabilization of PAK1 is independent of its catalytic activity and p21 GTPase binding capacities. Ectopic expression of PAK1 variants impairs JNK but not NF-κB pathway, which in turn suppresses the promoter activation and transcription of MMP-9. Expression of other MMPs such as MMP-2, MMP-3, and MMP-14 as well as TIMP-1 is, in contrast, not affected by PAK1. We also characterized the differences of MMP-9 expression between human keratinocytes and dermal fibroblasts. For keratinocytes, TNF-α or IL-1 is sufficient to induce MMP-9, while mesenchymal cells such as dermal fibroblasts and hepatic stellate cells require type-I collagen as an additional factor which boosts JNK activity to maximally induce MMP-9.

## Results

### Cytokine-induced expression of MMP-9 by human skin is partially reconstituted by primary keratinocytes and dermal fibroblasts

**Figure 1:**
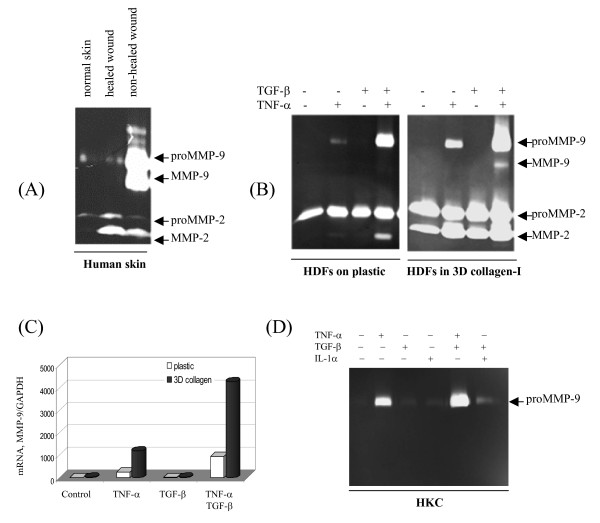
**Expression of MMP-9 by human skin of wounds is partially reconstituted by the skin cells**. (A) Gelatianses expression by human skin. Biopsies of normal skin, healed and non-healed wounds (full thickness, about 100 mg) were immersed in 1 ml DMEM for 6-hrs and secreted gelatinases were examined by zymography. (B) Early passages of human dermal fibroblasts (HDFs) were cultured on either plastic or in 3D type-I collagen, and treated by cytokines. After 3 days the conditioned medium was examined for gelatinases. (C) The mRNA levels of MMP-9 from HDFs cultured on plastic or in 3D type-I collagen with or without cytokines for 16 hrs were analyzed by real-time RT-PCR. (D) Zymography of the conditioned medium from the primary human keratinocytes (HKC) cultured for 3 days.

First, we surveyed the expression pattern of gelatinases by biopsies from patients with non-healed chronic (open wounds for more than 30 days) or healed wounds, as well as normal skin tissue. As shown in Fig. 1A, massive amount of proMMP-9 (92-kDa) and mature form (82-kDa) were evident in non-healed wounds, while minimal level of the proteinase was found in healed and normal skin tissues. Conversely, low level of proMMP-2 was evenly expressed by all three skin samples, while mature MMP-2 was found in both healed and non-healed skin. Excessive expression of MMP-9 in non-healed skin is associated with persistent inflammation in chronic wounds. To confirm the notion, isolated human dermal fibroblasts at the early passages (p < 3) were cultured on plastic, or embedded in 3-D type-I collagen. Cultured on plastic, dermal fibroblasts expressed a minimal level of proMMP-9 in response to TNF-α; under the combination of TNF-α and TGF-β, the fibroblasts produced massive proMMP-9 (Fig. 1B). Only in 3D type-I collagen, the fibroblasts also generated mature MMP-9 in response to the combination of TNF-α and TGF-β. We also measured the steady state of MMP-9 mRNA level. Dermal fibroblasts cultured on plastic or in 3D collagen gel were exposed to TNF-α, TGF-β, and their combination for 16 hrs, and the mRNA level of MMP-9 was determined by real-time RT-PCR. As shown, the mRNA gives almost identical ranking of expression as to the protein levels, demonstrating that the regulation of MMP-9 by TNF-α and collagen is mostly at the mRNA level (Fig. 1C). Thus, multiple extracellular cues including combination of two drastic different cytokines, TNF-α and TGF-β, and ECM render the dermal fibroblasts to generate maximal level of MMP-9, implying a situation in inflaming tissues.

Primary human keratinocytes produced minimal level of proMMP-9 in response to TNF-α, and the expression was also additionally enhanced in concert with TGF-β (Fig. 1D). Of note are two clear distinguished features between the dermal fibroblasts and keratinocytes in terms of MMP-9 expression. First, ECM has a profound role to induce MMP-9 expression by dermal fibroblasts but not keratinocytes (data not shown). Second, MMP-2, a mesenchymal MMP is largely absent in keratinocytes. Taken together, both the keratinocytes and dermal fibroblasts of human skin may contribute to the massive expression of MMP-9 under the stimulation of inflammatory cytokines in inflammation.

### PAK1 mediates TNF-α-induced expression of MMP-9 but not MMP-2

**Figure 2:**
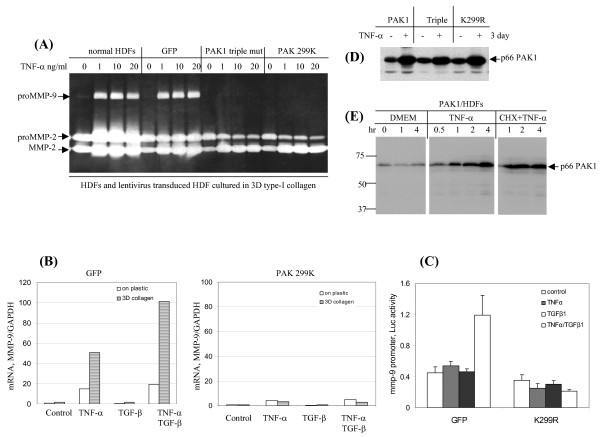
**PAK1 mediates TNF-α-induced MMP-9 but not MMP-2**. (A) HDFs and the cells transduced by lentivrius expressing GFP or PAK1 variants (K299R, catalytic inactive) were cultured in 3D type-I collagen. After culture for 3 days with TNF-α at the indicated concentrations, the gelatinases in the conditioned medium were measured by zymography. (B) HDFs expressing GFP or a PAK1 variant were cultured on plastic or in 3D type-I collagen and treated by TNF-α or/and TGF-β for 16 hrs. The mRNA of MMP-9 was measured by real-time RT-PCR and normalized by GAPDH. The results are representative of three independent experiments. (C) The activities of 670-bp 5'-promoter of human MMP-9 were measured by transient transfection of HDFs expressing GFP or PAK1 mutant (K299R) with pGL2 reporter plasmids encoding firefly luciferase and CMV promoter driving renilla luciferase. The resultant cells were stimulated by cytokines for 16 hrs (n = 6). (D) HDFs ectopically expressed PAK1 protein were stimulated by TNF-α for 3 days. PAK1 protein was measured by Western blot analysis. Of note is the increased the viral promoter driven PAK1 protein in response to TNF-α. (E) HDFs expressing PAK1 were treated with or without TNF-α together with cycloheximide (20 μg/ml). At the indicated intervals the cells were harvested for Western blot analysis of PAK1.

To elucidate the intracellular signaling to govern MMP expression we transduced human dermal fibroblasts with lentivirus to express GFP, PAK1 wild type, PAK1 triple mutant (kinase inactive and absence of p21GTPase binding), and PAK1 K299R mutant (kinase inactive) respectively. The viral transduced dermal fibroblastic cell lines were then embedded in type-I collagen, followed by TNF-α stimulation at the indicated dose for 3 days. Gelatinases in conditioned medium were resolved by zymography. Strikingly, the expression of proMMP-9 induced by the synergistic action of TNF-α and collagen was totally suppressed by PAK1 variants, but not GFP which works as a negative control (Fig. 2A). The specific effects of PAK1 on MMP-9 expression were evident by the absence of regulation on the expression of proMMP-2 as well as the maturation of the zymogen (transition from 72-kDa to 62-kDa). The wild-type PAK1 at the situation of over expression partially ablated MMP-9 expression, which may due to blockage of signaling traffics under over-expression (data not shown). Thereafter, we measured the steady state of MMP-9 mRNA by the human fibroblasts expressing either GFP or PAK1 mutant (K299R) cultured on plastic or in 3D type-I collagen. After 16 hr treatment by either TNF-α or TGF-β, as well as their combination, the MMP-9 mRNA was determined by real-time RT-PCR. As shown in Fig. 2B, induction of MMP-9 mRNA at all these conditions was totally impaired by the kinase inactive PAK1. Finally, we measured the effect of PAK1 on the 5'-promoter activity of the MMP-9 gene. The 5' promoter activities of MMP-9 were measured by dual luciferase assay through transfection of dermal fibroblasts with a reporter plasmid expressing firefly luciferase driven by a 670-bp 5'-promoter of MMP-9, while the control was monitored by renilla luciferase driven by CMV promoter. As shown in Fig. 2C the cytokine-induced activation of MMP-9 promoter was impaired by the PAK1 mutant.

### TNF-α-induced stabilization of the ectopically expressed PAK1 protein

We then addressed the nature of TNF-α regulation of PAK1. First, we analyzed the cell lines constitutively expressing PAK1 driven the viral promoter. After 3 days of TNF-α exposure the protein level of the ectopically expressed PAK1 was unexpectedly elevated, indicating a possible mechanism by which TNF-α signaling results in stabilization of PAK1 protein (Fig. 2D). Importantly, the TNF-α induced accumulation of PAK1 protein is independent of the kinase activities and p21 GTPase binding, as measured by the similar elevation of the variants of K299R and the triple mutant in response to TNF-α. Measurement at fine time points demonstrated again the prompt mode of elevation of the ectopically expressed PAK1 after TNF-α treatment (Fig. 2E). Because the ectopically expressed PAK1 is driven by constitutively active viral promoter, the TNF-α induced elevation of the kinase is, therefore, likely mediated by stabilization of the protein. To strengthen the notion we treated the dermal fibroblasts with cycloheximide to block protein synthesis and to monitor the degradation of the kinase. Under 20 μg/ml of cycloheximide, which sufficiently suppresses many protein syntheses, the PAK1 protein was still elevated under TNF-α treatment, demonstrating that the cytokine-dependent elevation of PAK1 is mediated by stabilization (Fig. 2E).

**Figure 3:**
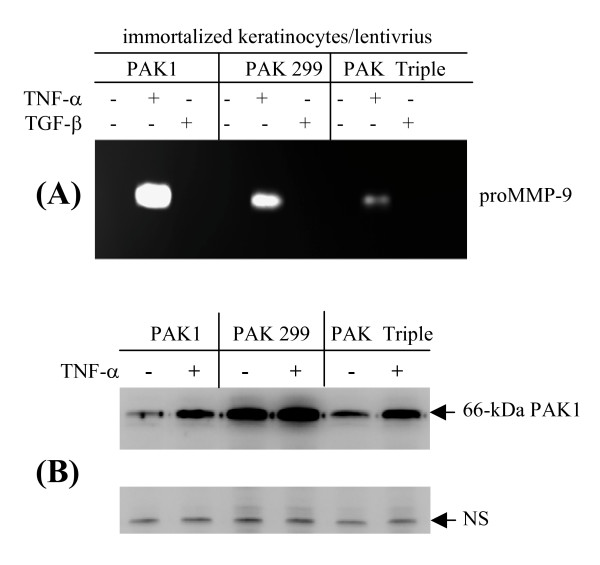
**TNF-α-induced expression of MMP-9 through PAK1, and stabilization of the ectopically expressed kinase by human keratinocytes**. Immortalized human keratinocytes transduced by lentivrius expressing GFP or PAK1 variants were cultured in KGM. After culture for 3 days with cytokines the gelatinases in the conditioned medium were measured by zymography. (B) PAK1 and a non-specific protein as a negative control were measured by Western blot analysis.

Immortalized human keratinocytes (IKC) were also transduced by the same set of lentiviruses, and the cells were treated by cytokines for 3 days. As shown in Fig. 3A, proMMP-9 was abundantly induced by IKC transduced by the wild type PAK1 virus in response to TNF-α but not TGF-β stimulation. Conversely, the TNF-α induced expression of proMMP-9 was partially abolished by the cells expressing the kinase-negative PAK1 variants. Similar to the fibroblasts, the protein levels of the wild type PAK1 in the keratinocyte was clearly enhanced by TNF-α treatment (Fig. 3B). Taken together, we demonstrated a novel regulation of PAK1 by TNF-α, which is mediated by an unknown mechanism to stabilize the PAK1 protein.

### Expression of MMP-9, but not MMP-2, -3, -14, is specifically controlled by PAK1

**Figure 4:**
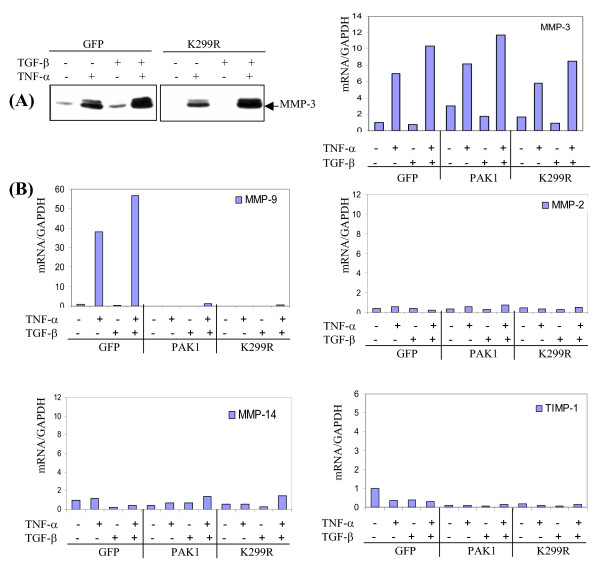
**MMP-9, but not other MMPs, is specifically regulated by PAK1**. (A) HDFs transduced by lentivrus expressing GFP or PAK1 variant (K299R) were embedded in type-I collagen and treated by cytokines for 3 days. MMP-3 in the conditioned medium was measured by Western blot analysis. (B) After 16 hrs of treatment with cytokines, the mRNA of MMPs and TIMP-1 were measured by real-time RT-PCR, normalized by GAPDH (n = 3).

The intriguing regulation of MMP-9 by PAK1 as shown in Fig. 2A prompted us to examine regulation of other MMPs. Dermal fibroblasts transduced by lentivirus expressing GFP or inactive PAK1 (K299R) were embedded in 3D type-I collagen. After 3 days of stimulation with cytokines, the secreted MMP-3 in conditioned medium was measured by Western blot analysis. As shown in Fig. 4A, TNF-α, but not TGF-β, induced MMP-3, while combination of the two cytokines promoted synergistic induction of MMP-3, in a manner very similar to MMP-9 expression. However, in a drastic contrast to MMP-9, the cytokine-induced MMP-3 expression is not affected by over expression of PAK1 variants. The mRNA levels of MMP-2, -3, -9, -14, and TIMP-1 were measured by real-time RT-PCR. As shown in Fig. 4B, after 16 hr stimulation the mRNA of MMP-3 was induced 6 folds by TNF-α and additionally enhanced by the combination with TGF-β, which is closely correlated to MMP-3 protein expression. The cytokine-mediated induction of these MMPs was not altered by forcedly expressed PAK1. In a similar fashion, the low level expression of the mRNA of MMP-2, -14, and TIMP-1 was also not altered by PAK1. Again, PAK1 variants thoroughly suppressed the cytokine-exerted MMP-9 mRNA elevation. These results clearly demonstrate a specific mode of PAK1 in regulation of MMP-9 expression.

### JNK, but neither p38 MAP kinase nor PI3 kinase, is downstreamof PAK1 to promote MMP-9 expression

**Figure 5:**
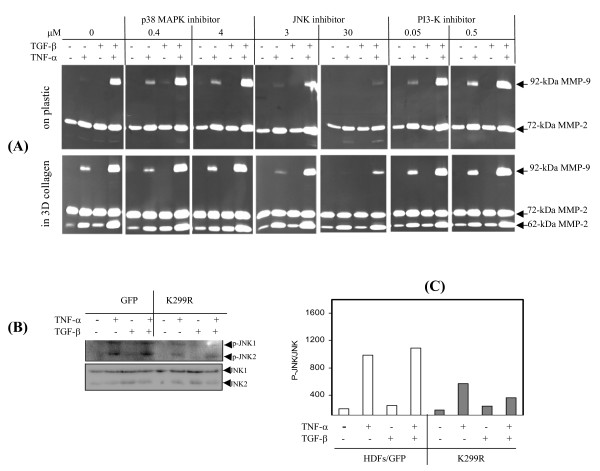
**JNK, as a downstream effector of PAK1, is essential for MMP-9 but not MMP-2 expression**. (A) Normal HDFs, cultured on plastic or in 3D type-I collagen, were treated by cytokines together with inhibitors for p38 MAP kinase, JNK, and PI-3' kinase respectively at their effective concentrations. MMP-9 and -2 in the conditioned medium were detected by zymography. (B) HDFs transduced by lentivirus expressing GFP or PAK1 variant (K299R) were stimulated by cytokines for 20 min. JNK1/2 and the phosphorylated JNK were measured by Western blot analysis. (C) The levels of phosphorylated JNK1/2 were quantitated by densitometry analysis, and normalized by their protein levels. Data are the averages of three experiments.

JNK signaling has been well defined as a key regulator to initiate the transcription of many MMPs including MMP-9 [20,21]. To probe the possible role of JNK in cytokine-induced MMP-9 expression, we treated human dermal fibroblasts in 3D type-I collagen or on plastic by inhibitors for JNK (SP600125), p38 MAP kinase (SB239063), and PI-3 kinase (wortmanin) at the effective concentration suggested by the manufacture. After 3 days of culture with TNF-α and/or TGF-β, the conditioned medium was examined for gelatinase activities. As shown in Fig. 5A, the cytokine-induced MMP-9 was suppressed thoroughly by the JNK inhibitor, although the inhibition efficacy was slightly lower by the cells cultured in 3D gel, which may be due to the adsorption or trap of the compound in the scaffold of ECM. Expression of proMMP-2 (72-kDa) and maturation to the active form (62-kDa) were clearly not altered by the JNK inhibitor, indicating specific inhibition and absence of general cellular toxicity. In contrast, the inhibitors for p38MAP kinase and PI3 kinase were found without effects on MMP-9 expression. Activation of JNK by fibroblasts ectopically expressing PAK1 variants was measured by antibody against p46 and p54 SAPK/JNK dually phosphorylated at Thr183 and Tyr185 (Fig. 5B). After 20 min treatment with cytokines JNK was phosphorylated in response to TNF-α by the controlled cells (GFP), and largely abrogated by the cells expressing inactive PAK1 (K299R) (Fig. 5C).

**Figure 6:**
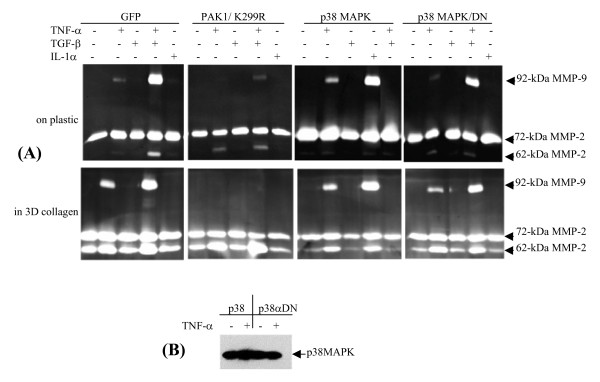
**p38 MAP kinase is not required for MMP-9 expression and stability of the kinase is not regulated by TNF-α**. (A) HDFs transduced by lentivirus expressing GFP, PAK1 (K299R), p38 MAP kinase, and its dominant negative one were cultured on plastic or in 3D type-I collagen, followed by stimulation by cytokines. After 3 days, MMP-9 and -2 in the conditioned medium were measured by zymography. (B) Protein of p38MAP kinase was measured by Western blot analysis.

In addition to JNK and NF-κB pathways, TNF-α also activates p38MAP kinase, which prompted us to examine its role in regulation of MMP-9 expression. Human dermal fibroblasts transduced by lentivius expressing p38MAP kinase or the dominant negative variant; and the resultant cells were cultured either on plastic or in 3D gel followed by treatment with cytokines. As shown in Fig. 6A, cytokine-mediated proMMP-9 expression was not altered by p38MAP kinase, which is in line with the results of the inhibition experiment (Fig. 5A). In contrast, MMP-9, but not MMP-2 expression, was totally suppressed by inactive PAK1. Moreover, protein stability of the ectopically expressed p38MAP kinase was not regulated by TNF-α (Fig. 6B). Thus, JNK1 is likely to be a downstream effector of PAK1 as demonstrated previously by others [22], and we showed here such moiety in the TNF-α signaling to control MMP-9 expression.

### Not regulated by PAK1, NF-κB signaling is essentialfor MMP-9 expression

**Figure 7:**
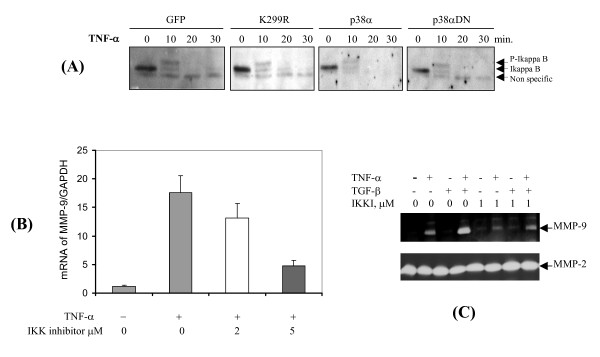
**NF-κB pathway is required for MMP-9 expression, but is independent of PAK1 and p38 MAP kinase**. (A) HDFs transduced by lentivrius expressing GFP, PAK1 (K299R), p38 MAP kinase, and its dominant negative ones were cultured on plastic and treated by TNF-α for the indicated intervals. Degradation of I-kappaB was measured by Western blot analysis. (B) HDFs were treated by TNF-α with IKK inhibitors at the indicated concentration for 6 hrs. The mRNA of MMP-9 was measured by real-time RT-PCR analysis. Results are from the average of three experiments. (C) HDFs were treated by TNF-α with IKK inhibitors at low concentration for 3 days. MMP-9 and -2 were measured by zymography.

Many studies have demonstrated the role of NF-κB in regulation of MMPs including MMP-9 by directly recruitment of NF-κB to the cis-elements in the 5'-promoter of MMP-9 [20,21]. Of interest is to know if NF-κB signaling is also under control by PAK1. Activation of NF-κB was measured by degradation of its inhibitor, IkappaB-α by the fibroblasts under the context of GFP, PAK1 (K299R), p38MAP kinase and its dominant negative variant. After 10 min of stimulation with TNF-α, IkappaB-α was promptly up-shifted, presumably through hyper-phosphorylation (Fig. 7A). At 20 min after the challenge the IkappaB-α was totally diminished by all these cells. Thus, in contrast to JNK, NF-κB is independent of PAK1 signaling. To confirm the notion of requirement of NF-κB we treated the fibroblasts with inhibitor for IKK, and measured the mRNA and protein of MMP-9. As shown in Fig. 7B and 7C, the cytokine-induced expression of MMP-9 at both mRNA and protein levels was partially suppressed by the IKK inhibitor. Conversely, expression of MMP-2 was not affected by the inhibitor. Thus, NF-κB signaling is required, but not under control by PAK1, to induce MMP-9 expression.

### Phosphorylation, activation, and stabilization of endogenous PAK1 in response to TNF-α/IL-1α

**Figure 8:**
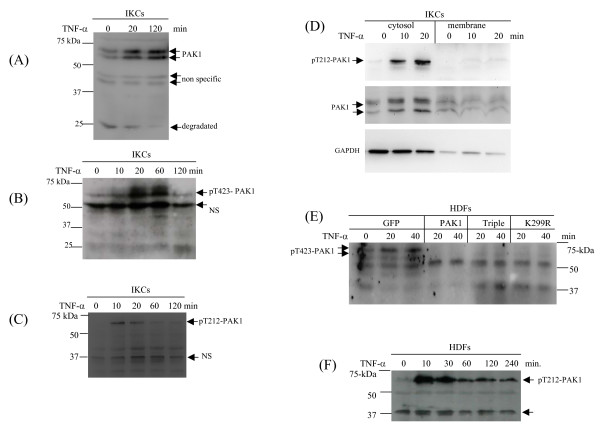
**Activation and stabilization of endogenous PAK1 in response to TNF-α by keratinocytes and dermal fibroblast**. (A) Keratinocytes (IKCs) were treated by TNF-α for the indicated periods and PAK1 protein was measured by Western blot analysis. Intact PAK1 and the putative degradation and non-specific bands were indicated. (B) Threonine-423 phosphorylation of PAK1 by the IKCs in response to TNF-α was measured by Western blot analysis. (C) Threonine-212 phosphorylation of PAK1 by the IKCs in response to TNF-α was measured by WB analysis. (D) Distribution of phosphorylated PAK1 in cytosolic and membrane compartments was measured by Western blot analysis. GAPDH was used as an indicator for cytosolic proteins. (E) Threonine-423 phosphorylation of PAK1 by HDFs was measured by Western blot analysis. (F) Thronine-212 phosphorylation by HDFs was measured by WB analysis.

Given the evidence of stabilization of the ectopically expressed PAK1 by TNF-a signaling, and regulation of MMP-9 expression by the kinase, an immediate question is, therefore, to what extend the endogenous PAK1 is actually activated and stabilized in response to TNF-α. As shown in Fig. 8A, the doublet bands of endogenous PAK1 were immediately elevated after 30 min treatment of keratinocytes by TNF-α, and the PAK1 protein was substantially accumulated at 120 min. A band at 25 kDa, also recognized by the antibodies for PAK1, a presumable degradation product at basal state, was gradually diminished, whereas the full length PAK1 accumulated after TNF-α treatment, indicating a possible mechanism that TNF-α suppresses the turnover of PAK1. As an internal control, two bands at about 45-kDa were constitutively expressed in a mode independent of TNF-α. As a general way to activate a protein kinase, a site within the pseudo-substrate loop in the catalytic domain is phosphorylated in response to stimulation [23]. Phosphorylation of PAK1 at threonine-423 has been demonstrated as an indicator for activation state of the kinase [24]. In response to TNF-α, phosphorylation of threonine-423, as measured by antibodies for the phosphopeptide, promptly appeared at 10 min, and peaked at 20 min (Fig. 8B). PAK1 can also be phosphorylated at threonine-212 presumably by Cdc2/Cdk5 [25]. As shown in Fig. 8C, threonine-212 phosphorylation occurred maximally at 10 min after TNF-α stimulation, prior to the threonine-423 phosphorylation.

**Figure 9:**
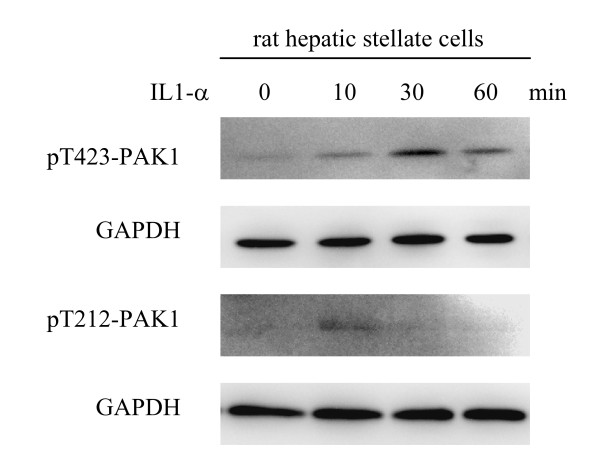
**IL-1 stimulation of PAK1 phosphorylation by primary rat hepatic stellate cells**. Primary rat hepatic stellate cells were stimulated by IL-1α for the indicated time. Phosphorylation of PAK1 at threonine 423 and threonine-212 and GAPDH were measured by Western blot analysis.

We then fractioned cellular compartments, and found that most of PAK1 protein and the threonine-212 phosphorylated version being present in cytosolic fractions rather than in the membrane pools. Similarly, threonine-423 of PAK1 was phosphorylated in dermal fibroblasts in response to TNF-α stimulation but in the prompt manner as in the keratinocytes (Fig. 8E). Importantly, fibroblasts over expressing PAK1 variants failed to show threonine-423 phosphorylation, which sufficiently explains its role in the impairment of the signaling in MMP-9 expression (Fig. 8E and 2). Similar to keratinocytes, dermal fibroblasts expressing GFP showed prompt phosphorylation of threonine-212 in response to TNF-α (Fig. 8F). We were interested to know whether PAK1 can be activated by IL-1, which shares many signaling pathways with TNF-α. Our previous work showed that the hepatic stellate cells can vigorously produce both proMMP-9 and the mature proteinase in response to IL-1 stimulation only by the cells cultured in type-I collagen [7,26]. As expected, in the rat hepatic stellate cells, PAK1 underwent phosphorylation at threorine-212 (10 min), followed by phosphorylation at threorine-423 (30 min) (Fig. 9). Taken together, through analysis of these three cell types we found that under TNF-α stimulation PAK1 is phosphorylated in a dynamics starting at threonine-212 followed by threonine-423 phophorylation, which confers the kinase to an active state. Given the fact of failed activation of PAK1 by the variants, and the evidence of their equal stabilization by these PAK1 variants, the TNF-α induced stabilization of PAK1 is, therefore, very likely independent of the kinase activation mechanism.

### Contribution of collagen to TNF-α-induced MMP-9 expression by fibroblasts is associated with persistent activation ofJNK

**Figure 10:**
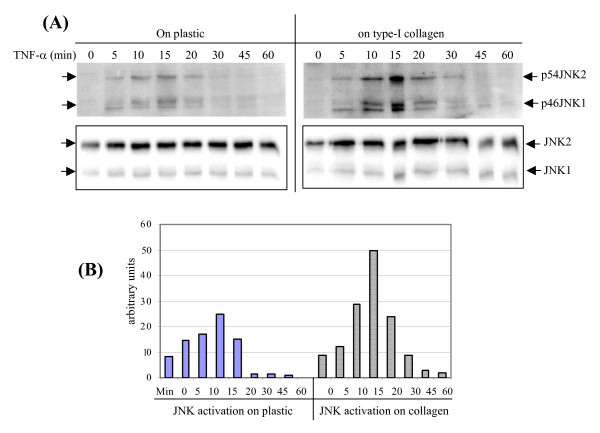
**Type-I collagen enhances TNF-α induced activation of JNK by dermal fibroblasts**. (A) HDFs were cultured on plastic or type-I collagen and stimulated by TNF-α for the indicted intervals. Phospho-JNK and JNK proteins were measured by Western blot analysis. (B) Activation of JNK was quantitated by densitometry analysis and plotted by the ratio of phosphor-JNK vs. JNK protein as arbitrary units.

It still remains outstanding how ECM boosts the TNF-α signaling to induce MMPs by the mesenchymal cells in tissue environment, as shown in Fig. 1. First, we examined whether collagen can boost the JNK signaling, for the reason, in part, of its role in MMP-9 regulation, and under the PAK1 signaling. Our previous work also demonstrated additive stimulation of the minimal promoter of human MMP-9 by collagen and TNF-α, while the convergences between the TNF-α and collagen signals are not known [6]. Thus far, we examined the influence of collagen on the kinetics of JNK activation by human dermal fibroblasts. Cells were cultured on plastic or in type-I collagen to measure the strength and speed of the response. As shown in Fig. 10, phosphorylation of both JNK1 and JNK2 promptly occurred at 5 min after TNF-α challenge, reached to peaks at 15 min, and followed by gradual decline. Although collagen alone could not trigger JNK activation, it did magnify the amplitude of JNK1/2 phosphorylation. Densitometry scanning revealed a two-fold enhancement of phospho-JNK1/2 by the cells cultured in type-I collagen. Therefore, the synergistic effect of collagen to cooperate with TNF-α in induction of MMP-9 can be explained, at least in part, by the amplification of JNK activities.

## Discussion

Expressed in early embryonic development, MMP-9 is largely silent in many adult tissues; while MMP-2, another member of the gelatinase family, is constitutively expressed in health tissues. However, upon injury such as mechanical trauma, thermal burn, and viral infection, MMP-9 is promptly elevated, indicating its role in wound healing by activating the stromal cells, releasing growth factors, and initiating cell migration. On the other hand, persistent presence of large amount of MMPs is often associated with, if not results in, many inflammatory diseases such as chronic wounds, arthritis, and cancer metastasis. Despite large body of efforts to discover the intracellular signal pathways to control MMPs in variety of cells, little is known as to how ECM in cooperating with the cytokine signals regulates MMP expression, particularly by the mesenchymal cells. In ECM scaffolds the mesenchymal cells are intensified by the intracellular cytoskeletal networks which are essential for cell anchoring, migration, and proliferation in the waves of cytokines during wound healing processes. Organization of intracellular cytoskeletal framework is largely orchestrated by small GTPases and their down stream effectors. To these regards, we investigated the role of PAK1 in regulation of MMPs by three cell types from msenchymal to epithelial cells. In summary, we have found (1) TNF-α triggers a sequential phosphorylation events in PAK1, starting at threonine-212 followed by threonine-423, which may confers the kinase into active state; (2) TNF-α promptly elevates PAK1 protein level, presumably through stabilization, which is independent on the kinase activity and p21 GTPase binding capacity; (3) TNF-α induced expression of MMP-9 is mediated by PAK1/JNK pathway which controls transcriptional initiation of MMP-9 promoter.

Originally identified as targets by GTP-loaded p21 protein, PAK family with 6 members has been demonstrated to serve as important regulators in cytoskeletal dynamic and cell motility, presumably through phosphorylation of the downstream substrates such as Lim kinase and myosin light chain kinase [12]. Like many protein kinases, PAK1 activity is restrained in latency by formation of homodimer through the interaction between the kinase inhibitory domain and the catalytic activation loop, which prevents the kinase from access the substrates [27]. The first step to provoke activation of PAK1 is believed to disrupt such trans-inhibition interaction, which can be conducted by variant ways such as binding of p21 GTPase, partial cleavage by caspase, association with sphingolipids, and phosphorylation [28]. The second step is to maintain the "opened" status by auto-phosphorylation at theronine-423 of the active loop in catalytic domain [29,30]. On the other hand, it is unknown whether inflammatory cytokines regulate PAKs, and how such regulation controls MMP in inflammation process.

In this study we first assessed the contribution of dermal fibroblasts and keratinocytes to the massive expression of MMP-9 by non-healing skin tissues. Although both dermal fibroblasts and keratinocytes produce MMP-9 in response to TNF-α/IL-1, dermal fibroblasts require type-I collagen as additional factor to maximally induce the proteinase, which makes pathophysiological sense as a paradigm between an enzyme and its substrate in a mutual demanding in order to maintain tissue homeostasis (Fig. 1). Still unknown is how TGF-β in concert with TNF-α enhances MMP-9 expression by fibroblasts of human and rodent skin, while TGF-β alone is not sufficient to induce the proteinase [6]. According to present results, TGF-β is unable to cooperate with TNF-α to activate JNK or NF-κB pathway (Fig. 5B and data not shown). However, the magnified activation of JNK by the dermal fibroblasts cultured on type-I collagen as shown in Fig. 10 may explain, at least in part, the role of TGF-β through the capacious capacity to induce of type-I collagen (data not shown). The role of TGF-β is surely cell-type specific and depends on the context of other signaling, as we found TGF-β suppresses the IL-1-induced expression of MMPs by hepatic stellate cells [7,26].

Still intriguing is how MMP-9, but not MMP-3, is specifically subjected to PAK1 control under the same TNF-α treatment (Fig. 2 and 4). The suppression of MMP-9 expression by PAK1 can be attributed, in part, to the transcription activation, since the ectopically expressed PAK1 mutant impairs the proximal 5'-promoter activities of MMP-9, and completely suppresses the accumulation of the mRNA and protein of MMP-9. Such suppression is in line with the impaired activation of JNK, but not NF-κB (Fig. 5 and 8). This notion is further reinforced by our inhibition experiment, by which JNK inhibitor thoroughly disrupts expression of MMP-9 (Fig. 5). Clues of PAK1 in regulates MAP kinase come from the work on yeast mating as well as reconstitution experiments [16,17,31,32]. In addition to JNK, PAK1 has been shown to induce activation of p38 MAP kinase pathways [33,34]. In our study, neither expression of p38 MAP kinase variants nor inhibition with specific inhibitor for the kinase does affect the MMP-9 at all (Fig. 4 and 7).

It is unknown, to date, how TNF-α promotes activation of PAK1 protein [35]. Phosphorylation of thronine-423 in the active loop has been demonstrated as an indicator for activation of PAK1 [15,24]. In addition to p21 GTPase binding and thereafter induced auto-phosphorylation of PAK1, PDK was also found to phosphorylate thronine-423 of PAK1 [15]. In this study we found TNF-α exerts sequential phosphorylation of PAK1, started at thronine-212 (10-min) and followed by thronine-423 phosphorylation (20-min). Following PAK1 activation is the accumulation of the protein of the kinase per se. We concluded that TNF-α induced accumulation of PAK1 is regulated at post-transcriptional level. Such conclusion is based on the following evidences: (1) Cycloheximide, which suppresses protein synthesis in general, does not affect PAK1 protein under TNF-α stimulation; (2) The constitutively expressed PAK1 under viral promoter is also up regulated by TNF-α, and as a control the p38 MAP kinase is not altered at all, which strongly supports the mechanism of protein stabilization; (3) Up regulation of PAK1 by TNF-α or IL-1 seems ubiquitous, since it is detected by human dermal fibroblasts, and keratinocytes, as well as rat hepatic stellate cells; (4) TNF-α induced accumulation of 66-kDa PAK1 is inversely correlated with the loss of the degradation band at 25-kDa (Fig. 8A). All these indicate that TNF-α may somehow attenuate the degradation of PAK1 protein. Clearly, the TNF-α induced stabilization of PAK1 is independent of its intrinsic kinase and p21 GTPase-binding capacity. Most of PAK1 is compartmented in cytosolic pool, of which is promptly induced by TNF-α; while the kinase in the membrane fractions is not regulated (data not shown). To date, it is unknown how TNF-α regulates PAK1 stability.

## Conclusion

We identified a novel mechanism of MMP-9 expression controlled by TNF-α through stabilization and activation of PAK1 which in turn activates JNK pathway leading the transcription of MMP-9.

## Methods

### Materials and reagents

Cytokines were purchased from R&D Systems. Antibodies against PAK1, p38 MAP kinase, I-kappaB, MMP-3 and MMP-9 were purchased from Santa Cruz Biotechnologies. Antibodies for JNK, phospho-JNK (Phospho-SAPK/JNK (Thr183/Tyr185), phospho-PAK1 (Thr423)/PAK2 (Thr402) were from Cell Signaling Technology. Antibodies for phosphor-212-threonine-PAK1 were from Sigma. Antibodies for GAPDH were from Chemicon. SuperSignal West Fermto Maximum Sensitivity Substrate was from PIERCE. The 5'-670 bp promoter of human MMP-9 was cloned into plasmid pGL2/firefly luciferase (pGL-5'670-MMP9). The Dual-Luciferase^® ^Reporter (DLR) Assay System was from Promega. Lentivirus encoding PAK1 variants including wild type, triple mutant (H83L, H86L and K299R) and K299R were kindly provided by G. Bokoch (The Scripps Research Institute, La Jolla, CA). Collagenase, inhibitors for IKK (#401486), JNK inhibitors (SP600125), and p38 MAP kinases (SB 239063) were from Calbiochem.

### Biopsies

Clinical biopsies including normal skin and chronic wounds were collected according to the protocol approved by Internal Review Board at the University of Southern California and are consent by patients. The 6-mm punch biopsies were placed in 2-ml DMEM with antibiotics (200 U/ml penicillin G sodium, 200 U/ml streptomycin sulfate and 0.5 μg/ml amphotericin B). To accumulate secreted factors the biopsies were incubated in the medium for 6 hours with supply of 5% CO_2 _at 37°C. The conditioned medium was cleared of debris by centrifugation at 5,000 g prior to zymography or Western blot analysis.

### Cell culture, lentiviral transduction, and luciferase assay

To isolate human dermal fibroblasts, full thickness skin was treated by 20 mM EDTA in DMEM at 37°C for 3 hrs. After removal of epithelial sheets the dermal tissue was incubated in collagenase (2 mg/ml) in DMEM at 37°C for 16 hrs. The resultant cells were seeded on dishes and cultured in DMEM with 10% FBS. The second passage of cells was used for experiments. Immortalized human keratinocytes were kindly provided by Dr. David Woodley at USC. Primary rat hepatic stellate cells were supplied from the USC Research Center for Alcoholic and Pancreatic Diseases, and cultured in DMEM with 10% FBS. Kratinocytes and human dermal fibroblasts were transduced by lentivirus prepared in 293T cells. To ensure the high transducing efficiency the cells were infected twice. Efficiency of transduction was measured by immunostaining with anti-PAK1 as well by expression of green fluorescent protein as an indicator. To create 3-dimensional culture, fibroblasts were embedded in type-I collagen as previously described [6]. To measure the promoter activities the dermal fibrobalsts were transfected by a reporter plasmid together with pGL/CMV-renilla luciferase as a reference. Luciferase activities were measured by the dual luciferase assay kit.

### Cell treatment and real-time RT-PCR

Cells on plastic or 3D ECM were treated with TNF-α (10 ng/ml), IL-1α (10 ng/ml), TGF-β1 (1 ng/ml) in DMEM with 1% FBS for 16 hrs. Total RNA was extracted using TRIZOL reagent according to the manufacturer's instructions (Invitrogen Life Technologies). First-strand cDNA was produced using First-Strand cDNA Synthesis by SuperScript II Reverse Transcriptase with random primers. Two micrograms of total RNA was used for each reverse transcription reaction mixture (20 μl). Real-time PCR was carried out using an ABI Prism 7900 HT (Applied Biosystems). 10 μl reactions were set up in 384-well PCR plate using the following final concentrations: 1 μmole each of forward and reverse primers, 1× SYBR Green master mix (qPCR Mastermix Plus for SYBR Green I, Eurogentec), and 5 ng of cDNA. For each condition three duplicates were used to minimize the variation. Cycling conditions were as follows: initial step (50°C for 2 min), hot activation (95°C for 10 min), amplification (95°C for 15 s, 60°C for 1 min) repeated 40 times, and quantification with a single fluorescence measurement. Data were analyzed using ABI Prism SDS 2.1 software. The relative gene expression was calculated by the Δ*Ct *method. Briefly, the resultant mRNA was normalized to its own GAPDH. Final results were expressed as *n*-fold difference in gene expression relative to GAPDH mRNA and calibrator as follows: *n*-fold = 2^-(Δ*Ct *sample-Δ*Ct *GAPDH)^, where Δ*Ct *values of the sample and the calibrator were determined by subtracting the average *Ct *value of the transcript under investigation from the average *Ct *value of the GAPDH gene for each sample. For human MMP-9 the forward primer has the sequence GGG AGA CGC CCA TTT CG and the reverse primer is CGC GCC ATC TGC GTT T. For GAPDH, the forward primer, GAA GGT GAA GGT CGG AGT 3', and backward primer GAA GAT GGT GAT GGG ATT TC 3' 20 mer. For MMP-14, the primers are TGG AGG AGA CAC CCA CTT TGA, and GCC ACC AGG AAG ATG TCA TTT C. For MMP-2, the primers are GAG AAC CAA AGT CTG AAG AG, and GGA GTG AGA ATG CTG ATT AG. For MMP-3, the primers are GCT GCA AGG GGT GAG GAC AC, and GAT GCC AGG AAA GGT TCT GAA GTG. The primers for TIMP-1 are TCT GGC ATC CTC TTG TTG CTA T, and CCA CAG CGT CGA ATC CTT.

## Authors' contributions

LZ carried out the cell culture, zymography, Western blot, and real-time PCR experiments. CY did luciferase assay, lentivirus preparation, and cell culture. YK did the Western blot for hepatic stellate cells. WL was a collaborator for viral vector and RG helped for cell culture. WG conceived the study, and participated in biopsy. YPH directed the whole study, participated in the experimental design and wrote the manuscript.
